
JOURNAL INFORMATION
==============================
NLM Title Abbreviation: Wirtschaftsdienst
Journal ID: Wirtschaftsdienst
NLM Journal ID: 101086612

Wirtschaftsdienst (Hamburg, Germany : 1949)

ISSN: 0043-6275

ARTICLE INFORMATION
==============================
PMCID: PMC8052197
PMID: 33897055
DOI: 10.1007/s10273-021-2892-7
Article ID: 2892
Article version: 1
Subjects: Zeitgespräch

Industrialisierung im Deutschen Reich: Welche Rolle spielte die öffentliche Infrastruktur?

Hornung Erik hornung@wiso.uni-koeln.de

grid.6190.e 0000 0000 8580 3777 Wirtschafts- u. Sozialwissensch. Fakultät, Universität zu Köln, Albertus-Magnus-Platz, 50923 Köln, Deutschland
Electronic publication date: 2021 Apr 17
Print publication date: 2021
Volume: 101
Issue: 4
First page: 258
Last page: 262
Copyright: © Der/die Autor:in(nen) 2021
License: Open Access: Dieser Artikel wird unter der Creative Commons Namensnennung 4.0 International Lizenz veröffentlicht (https://creativecommons.org/licenses/by/4.0/deed.de).
Open Access wird durch die ZBW — Leibniz-Informationszentrum Wirtschaft gefördert.
License URL: https://creativecommons.org/licenses/by/4.0/

issue-copyright-statement: © The Author(s) 2021

==============================

In der wirtschaftspolitischen Diskussion sind die schwache öffentliche Investitionstätigkeit und der Zustand der Infrastruktur in Deutschland seit Jahren ein wiederkehrendes Thema (Clemens et al., 2021). Dabei wird der Schuldenbremse eine substanzielle Rolle für den Investitionsstau zugeschrieben, der sich unter anderem in mangelnder Erneuerung der Verkehrsinfrastruktur und mangelnden Investitionen in ein zukunftsfähiges Bildungs- und Gesundheitswesen widerspiegelt. Die aktuelle COVID-19-Krise könnte zukünftig dazu führen, dass der Anstieg von Transfers an Haushalte und Unternehmen noch weniger Spielraum für öffentliche Investitionen lässt. Dabei zeigen Studien immer wieder, dass öffentliche Investitionen das langfristige Wachstum unterstützen, speziell dann, wenn sie gute Voraussetzungen für private Investitionen schaffen. Ein Blick in die Vergangenheit zeigt, dass positive Effekte von Infrastrukturinvestitionen in Deutschland eine lange Geschichte haben.

### Industrialisierung und Strukturwandel

Die Gründung des Deutsche Reichs im Jahr 1871 fiel in eine Phase tiefgreifenden Strukturwandels, demografischer Transformation, anhaltender Urbanisierung, wachsender Ungleichheit und Globalisierung. Die erste Phase der Industrialisierung, häufig mit dem Beginn des Eisenbahnbaus und der Gründung des Deutschen Zollvereins in den 1830ern assoziiert, ging mit einer innerdeutschen Marktintegration einher. Neben dem Eisenbahnbau kann in den historischen Berufszählungen eine stetig steigende Zahl von Beschäftigten im Bergbau und in zentralisierten Fabriken der Textil-, Metall und Maschinenindustrie beobachtet werden. Die zweite Phase der Industrialisierung begann in der zweiten Hälfte des 19. Jahrhunderts und nahm zur Zeit der Reichsgründung deutlich an Schwung auf. Diese Hochindustrialisierung war durch ein rasches Wachstum der chemischen und elektronischen Industrien und des Maschinenbaus gekennzeichnet – Branchen, in denen deutsche Unternehmen international die technologische Führerschaft übernahmen. Bei der Bereitstellung der benötigten Fachkräfte spielten möglicherweise sowohl das Bildungssystem als auch das Gesundheitssystem eine wichtige Rolle.

### Rolle der Eisenbahn in der Industrialisierung

Angefangen mit der ersten deutschen Bahnlinie zwischen Nürnberg und Fürth im Jahr 1835, wurden bis zum Ersten Weltkrieg im Deutschen Reich Eisenbahnstrecken mit einer Länge von ca. 60.000 km errichtet. Bei Reichsgründung waren es noch ca. 19.000 km gewesen.[1] Anfangs wurden Strecken in Preußen vornehmlich von privaten Eisenbahngesellschaften errichtet und betrieben, wohingegen Baden, Bayern und Württemberg weitestgehend eigene Staatsbahnen betrieben. Bei Reichsgründung herrschte allerdings in allen größeren Staaten ein Mischsystem aus privaten und staatlichen Bahnen. Eisenbahnpolitik blieb auch nach Reichsgründung überwiegend in der Verantwortung der verschiedenen Einzelstaaten. In den 1880er Jahren kam es zu einer Welle von Verstaatlichungen, vor allem der preußischen Privatbahnen, die sich aufgrund der guten Ertragslage verschiedener Strecken sehr positiv auf den Haushalt auswirkte. Es wurden nun aber auch weniger profitable Strecken gebaut, die Regionen mit geringerer Bevölkerungsdichte versorgten (Fremdling, 1983).

Durch Konzessionierung sowie Einschränkungen der Preis- und Fahrplangestaltung griffen die Staaten auch direkt in die Errichtung der Stecken und den Betrieb der privaten Eisenbahngesellschaften ein (Ziegler, 1996). Beispielsweise zielte die verfassungsmäßige Einführung des „Einpfennigtarifs“ darauf ab, die Beförderung von Kohle und Holz über größere Distanzen zu fördern und damit die Transportkosten für die wichtigsten Energieträger der heimischen Industrie zu reduzieren.

Tabelle 1
**Frachtsätze im nationalen Durchschnitt nach Transportart**
Frachtsätze in Pfennig pro Tonnenkilometer

| Jahr | See | Fluss | Schiene | Straße |
|---|---|---|---|---|
| 1846 | 1,61 | 4,50 | 11,20 | 40,00 |
| 1861 | 1.53 | 1,60 | 4,65 | 40,00 |
| 1875 | 1,21 | 1,55 | 3,42 | 40,00 |
| 1882 | 1,05 | 0,97 | 3,02 | 40,00 |

Quelle: Gutberlet (2013).

Die zentrale Rolle der Eisenbahn für die deutsche Industrialisierung ist unstrittig. Durch substanzielle Transportkostensenkungen und Nachfrageeffekte war sie wesentlich für das Wachstum in den Bereichen Kohle, Eisen, Stahl und Maschinenbau verantwortlich. Im Zeitraum von 1851 bis 1879 betrug der Anteil der Nettoinvestitionen, die durch den Eisenbahnbau generiert wurden, zwischen 10 % und 20 % (Fremdling, 1975).

Die Reduzierung von Transportkosten (vgl. Tabelle 1) spielte damals wie heute eine zentrale Rolle für das Wachstum des interregionalen Handels und für die Standortentscheidungen von Unternehmen. Neuere ökonometrische Studien bestätigen, dass die Verbindung mit einer Eisenbahnlinie in der Tat dazu führte, dass Märkte stärker integrierten. Nach Eröffnung einer Verbindung zwischen zwei Städten sank die Preisdifferenz von Weizen im Zeitraum 1820 bis 1880 um ca. die Hälfte (Keller and Shiue, 2014). Während die frühen Fabriken sich vornehmlich an Flüssen ansiedeln mussten, um ihre Maschinen mit Wasserkraft zu betreiben, konnten sich Fabriken nun wegen der niedrigen Transportkosten für Kohle verstärkt dort ansiedeln, wo es eine hohe Nachfrage nach ihren Produkten und ein hohes Angebot an Arbeitskräften gab. Die Loslösung von natürlichen Ressourcen führte entsprechend zu Agglomerationseffekten. Die durchschnittliche Firmengröße in preußischen Städten mit Eisenbahnanschluss war nahezu doppelt so groß wie in vergleichbaren Städten ohne Anschluss (Hornung, 2015). Preußische Städte profitierten nicht nur durch die Ansiedlung größerer Unternehmen, sondern auch durch eine höhere Zuwanderung – die Bevölkerung dieser Städte wuchs im Schnitt ca. 1 bis 2 Prozentpunkte pro Jahr schneller. Auch für Württemberg können regionale Wachstumseffekte der historischen Eisenbahnen nachgewiesen werden. Eine Gemeinde mit Eisenbahnanschluss wuchs demnach um 0,5 Prozentpunk-

1 In der Bundesrepublik Deutschland waren im Jahr 2020 noch ca. 38.400 km in Betrieb.

te schneller (Braun und Franke, 2019). Weitere Ergebnisse lassen zudem schließen, dass die Agglomerationseffekte zu höheren Arbeitseinkommen führten, die nur teilweise durch Zuwanderung ausgeglichen wurden, sodass beides sich in steigenden Immobilienpreisen niederschlug.

Neben den beschriebenen unmittelbaren Auswirkungen historischer Infrastrukturinvestitionen, finden sich in der Literatur immer mehr Hinweise auf langfristige Effekte. Die Entscheidung darüber, welche Verkehrsachsen ausgebaut werden sollen und den Vorzug gegenüber anderen erhalten, hat somit möglicherweise weitreichendere Konsequenzen als den Entscheidungstragenden bewusst ist. Es sollte beispielsweise berücksichtigt werden, dass Regionen, die besser miteinander verbunden werden, nicht nur mehr miteinander handeln, sondern eine stärkere Integration jeglicher Art vollziehen. Studien, die Integration über sehr lange Zeiträume, z.B. bis ins antike Rom verfolgen, zeigen, dass damals besser verbundene Regionen heute ähnlichere Präferenzen und Werte haben, was wiederum einen zusätzlichen positiven Effekt auf den Handel hat (Flückiger et al., 2019).

## Rolle der Bildung in der Industrialisierung

Im frühen 19. Jahrhundert waren die deutschen Staaten weltweit führend im Bereich Grundbildung. Die Schulbesuchsquoten in vielen deutschen Staaten lagen deutlich höher (vgl. Abbildung 1) als in England (1871 ca. 76 %), dem Land, das in der Industrialisierung führend war. Die deutsche Volksschulbildung wurde international bewundert und Modellschulen wurden von Delegationen aus den USA und England besucht. Bei Reichsgründung besuchten nahezu alle Kinder im schulfähigen Alter die Volksschule, die Geschlechterlücke war seit Jahren gering und in Preußen lag die Lese- und Schreibfähigkeit der Bevölkerung bei über 85 %.

Diese Werte wurden innerhalb eines strikt föderalen Systems erreicht. Jedes der 26 Länder innerhalb des Deutschen Reichs verwaltete seine Bildungsangelegenheiten selbst. Zwar ging die Reichsgründung mit einer Zentralisierung der Bildungssysteme der einzelnen Länder einher, Schulverwaltung und -finanzierung waren jedoch typischerweise lokal verankert. Die hervorragenden statistischen Daten der preußischen Zählungen zeigen, dass es schon Anfang des 19. Jahrhunderts weder an Infrastruktur noch an Lehrkräften mangelte, um die Nachfrage nach Volksschulbildung zu decken (Cinnirella und Hornung, 2016). Inhaltlich sahen die frühen Lehrpläne zwar die Vermittlung einer breiten Allgemeinbildung vor, scheiterten aber an der lokalen Schulwirklichkeit, insbesondere auf dem Land. Der typische Lehrplan in der ersten Hälfte des 19. Jahrhunderts konzentrierte sich ausschließlich auf das Rezitieren des Katechismus sowie auf Lesen, Schreiben und Singen. Nach Reichsgründung wurde die Schulaufsicht von der Kirche an den Staat übertragen, die Lehrerausbildung vereinheitlicht, und Teile des Religionsunterrichts wurden durch Unterricht in Naturwissenschaften und Turnen ersetzt.

Abbildung 1

**Schulbesuchsquote in der Volksschule in den größeren deutschen Staaten**

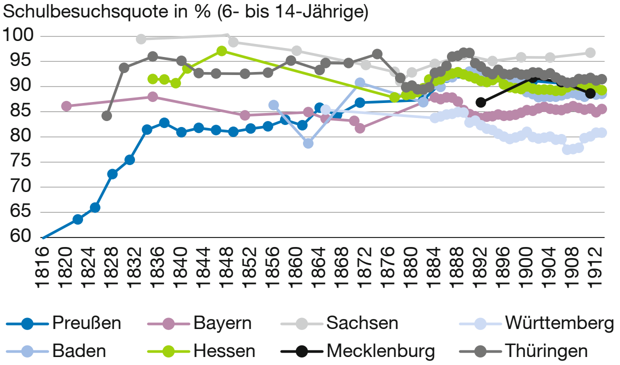

Quelle: Becker, Cinnirella und Hornung (2021).

Ein 1872 während des Kulturkampfs verabschiedetes Schulaufsichtsgesetz verlagerte die Befugnis zur Ernennung von Schulinspektoren von der Kirche auf den Staat mit dem Ziel, den Einfluss der katholischen Kirche zu beseitigen. Die Hauptaufgabe der Schulinspektoren bestand in der Überwachung des Schulbesuchs. Sie berichteten aber auch über die Leistungen der Schülerschaft und den Unterricht der Lehrkräfte. Die Einsetzung von staatlichen Inspektoren erhöhte die Schulbesuchsquote in den katholischen Regionen im Osten Preußens um ca. 6 %, hatte jedoch kaum Einfluss auf die katholischen Regionen im Westen Preußens (Schueler, 2018). Der Effekt lässt sich speziell durch eine Verringerung der Klassengrößen im Osten und einer hieraus resultierenden Verbesserung des Unterrichts erklären.

Der Beitrag des gut entwickelten Volksschulwesens für die Entwicklung und Industrialisierung Deutschlands sollte nicht unterschätzt werden. Industrien, in denen Deutschland verhältnismäßig rückständig war, profitierten von der breiten Grundbildung der Bevölkerung. Entsprechend der Theorie von Nelson und Phelps (1966), benötigt ein Land, je nach technologischer Entwicklung sowohl Massenbildung als auch Spitzenbildung. Je weiter eine Industrie von der technologischen Grenze entfernt ist, desto mehr kann sie von dieser Rückständigkeit profitieren, wenn sie neue Technologien von anderen Ländern übernehmen kann. Bei dieser Art der technologischen Diffusion ist es förderlich, wenn große Teile der Bevölkerung über eine breite Basisbildung verfügen und sich gut auf die neuen Produktionstechniken und -prozesse einstellen können. Im Vergleich zu England waren viele deutsche Industrien bei Reichsgründung technologisch unterentwickelt. Diese Industrien profitierten entsprechend vom guten Bildungsstand in Deutschland, als die englischen Technologien verfügbar wurden. Insbesondere während der zweiten Phase der Industrialisierung wuchsen die Beschäftigungszahlen in der Industrie dort stärker, wo die Lese- und Schreibfähigkeit der Bevölkerung besser ausgeprägt war (Becker, Hornung und Wößmann, 2011).

Für Industrien, die näher an der internationalen Technologiegrenze operieren, benötigt ein Land neben der Massenbildung auch Spitzenbildung, um wettbewerbsfähig zu sein und eigene Innovationen hervorzubringen. In Deutschland waren das die chemische und die elektrotechnische Industrie, deren Bedarfe nach Reichsgründung verstärkt von den Universitäten bedient wurden. Wie Abbildung 2 zeigt, kam es in der zweiten Hälfte des 19. Jahrhunderts zu einem starken Anstieg des Anteils der Studierenden im Bereich Naturwissenschaften und Mathematik, während z.B. der Anteil der Studierenden in Theologie sank. Universitäten waren allerdings auch schon in der ersten Hälfte des 19. Jahrhunderts förderlich für die Innovationstätigkeit in Deutschland (Dittmar und Meisenzahl, 2021). Andererseits waren hohe Schulbesuchsquoten in weiterführenden Schulen und Gymnasien keine Voraussetzung für die Innovationstätigkeit in Deutschland (Cinnirella und Streb, 2017). Diese Schulen konzentrierten sich damals eher auf die Vermittlung klassischer und humanistischer Inhalte. Gymnasien qualifizierten ihre Schülerschaft zwar für den Hochschulabschluss, in vielen Studienfächern führte der Abschluss jedoch zu einer Laufbahn in der öffentlichen Verwaltung.

Insgesamt lassen sich Angebot und Nachfrage von gut ausgebildeten Fachkräften im 19. Jahrhundert nur schwer auf die heutige Zeit übertragen. Je nach Industrie ist die qualifizierte Breitenbildung neben der Spitzenbildung auch heute noch ein Standortvorteil für deutsche Unternehmen.

## Rolle des Gesundheitswesens in der Industrialisierung

Die negativen Folgen der Industrialisierung waren insbesondere für die Arbeiterschaft zu spüren. Während der zweiten Hälfte des 19. Jahrhunderts rief die „soziale Frage“, die Verelendung der Arbeiter in den wachsenden Städten, nach immer mehr politischer Aufmerksamkeit. Die schlechten und unhygienischen Wohnverhältnisse in den Großstädten führten unter anderem zum Ausbruch von Epidemien und tiefgreifenden Problemen im Gesundheitswesen. Im Angesicht steigender Stimmanteile für die sozialdemokratischen Parteien, reagierten Reichskanzler Bismarck und das Deutsche Reich auf die Missstände mit der Einführung der Sozialversicherung. Im ersten Schritt wurde 1884 die Krankenversicherung für die Arbeiter eingeführt, die sich ausschließlich aus Beiträgen der Versicherten und ihrer Arbeitgeber finanzierte.

Abbildung 2

**Anteil der Universitätsstudierenden in Theologie und mathematisch-naturwissenschaftlichen Fächern**

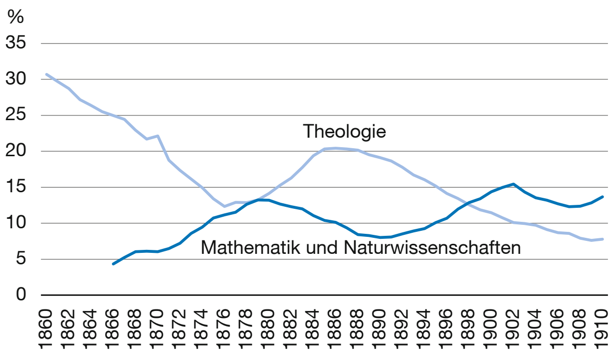

Quelle: Diebolt (2005).

Durch den Ausbau des Gesundheitswesens wurde die Lage deutlich verbessert. Im Zeitraum 1850 bis 1913 halbierte sich die Sterbeziffer nahezu und sank von ca. 27 pro 1.000 Einwohner auf ca. 15 pro 1.000 Einwohner. Für diese Veränderung war die Reduzierung der Säuglingssterblichkeit von ca. 225 pro 1.000 auf ca. 160 pro 1.000 maßgeblich. Die Krankenversicherung erklärt den Rückgang der Sterbeziffer im Zeitraum 1884 bis 1904 zu etwa einem Sechstel (Bauernschuster, Driva und Hornung, 2020). Die Krankenversicherung wirkte jedoch nicht durch Heilung, sondern durch Krankheitsprävention. Durch die Versicherung erlangte die Arbeiterschaft erstmals umfassenden Zugang zu Ärzten, die in den deutschen Universitäten in Hygienefragen gut ausgebildet waren und die neuen wissenschaftlichen Erkenntnisse zu ansteckenden Krankheiten direkt weitergeben konnten.

Ärzte spielten auch eine entscheidende Rolle hinsichtlich der Maßnahmen zur öffentlichen Gesundheitsfürsorge. Auf ihr Drängen investierten die deutschen Gemeinden nach Reichsgründung massiv in die Verbesserung der Trinkwasserzufuhr und der Abwasserentsorgung (vgl. Abbildung 3). Der Anteil von deutschen Städten mit einem Wasserwerk stieg zwischen 1870 und 1900 von 9 % auf 52 %. Während die verbesserte Wasserversorgung nur einen geringen Beitrag zur Verringerung der Sterblichkeit leistete, erklärt der Bau von städtischer Kanalisation den Rückgang der Sterbeziffer im Zeitraum 1877 bis 1913 zu etwa einem Fünftel (Gallardo-Albarran, 2020).

## Schlussbemerkung

Zusammenfassend kann man sagen, dass die hier beschriebenen Infrastrukturinvestitionen sich positiv auf die langfristige Entwicklung Deutschlands auswirkten. Natürlich sollte berücksichtigt werden, dass die damaligen Rahmenbedingungen sich grundsätzlich von den heutigen unterschieden und die Rolle der Wirtschaftspolitik

Abbildung 3
**Zahl der Inbetriebnahmen städtischer Kanalisation**

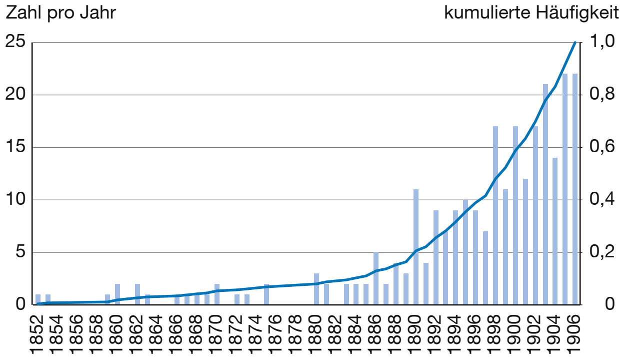

Quelle: Bauernschuster, Driva und Hornung (2020).

und des Staates in der Bereitstellung von Infrastruktur eine deutlich geringere war. Auch unterscheiden sich die heutigen Herausforderungen an Infrastrukturinvestitionen, wie Digitalisierung und Dekarbonisierung, grundlegend. Entsprechend gelten Implikationen für die heutige Wirtschaftspolitik nur bedingt und können nur unter Berücksichtigung des Kontextes auf die heutigen Begebenheiten übertragen werden.

Prof. Dr. Erik Hornung lehrt Wirtschaftsgeschichte an der Universität zu Köln.


Literatur

Bauernschuster S Driva A Hornung E Bismarck’s health insurance and the mortality decline Journal of the European Economic Association 2020 18 5 2561 2607 10.1093/jeea/jvz052
Becker S O Hornung E Woessmann L Education and catch-up in the industrial revolution American Economic Journal: Macroeconomics 2011 3 3 92 126
Becker, S. O., F. Cinnirella und E. Hornung (2021), Bildung, Entwicklung und Nationsbildung im 19. Jahrhundert, in U. Pfister, J.-O. Hesse, M. Spoerer und N. Wolf (Hrsg.), Deutschland 1871: Die Nationalstaatsbildung und der Weg in die moderne Wirtschaft, Mohr Siebeck.
Braun, S. T. und R. Franke (2019), Railways, Growth, and Industrialisation in a Developing German Economy, 1829–1910, MPRA Paper, 93644, University Library of Munich.
Cinnirella F Hornung E Landownership concentration and the expansion of education Journal of Development Economics 2016 121 135 152 10.1016/j.jdeveco.2016.03.001
Cinnirella F Streb J The role of human capital and innovation in economic development: evidence from post-Malthusian Prussia Journal of Economic Growth 2017 22 193 227 10.1007/s10887-017-9141-3
Clemens M Dullien S Fratzscher M Grimm V Hellwig M Hüther M Jung M Michelsen C Nöh L Rietzler K Schwarz M Tober S Nachhaltiges Wachstum: Braucht Deutschland ein Investitionsprogramm? Wirtschaftsdienst 2021 101 3 157 180 10.1007/s10273-021-2868-7 33746300 PMC7964466
Diebolt, C. (2005), Die langfristige Entwicklung des Schulsystems in Deutschland im 19. und 20. Jahrhundert, GESIS Datenarchiv, ZA8221 Datenfile Version 1.0.0.
Dittmar, J. und R. Meisenzahl (2021), The Research University, Invention, and Industry: Evidence from German History, mimeo, London School of Economics.
Flückiger, M., E. Hornung, M. Larch, M. Ludwig und A. Mees (2019), Roman transport network connectivity and economic integration, CEPR Discussion Paper, 13838, Centre for Economic Policy Research.
Fremdling, R. (1975), Eisenbahnen und deutsches Wirtschaftswachstum 1840–1879, Gesellschaft für Westfälische Wirtschaftsgeschichte.
Fremdling, R. (1983), Germany, in P. O’Brien (Hrsg.), Railways and the Economic Development of Western Europe, 1830–1914, 121–147, St. Martin’s Press.
Gallardo-Albarrán D Sanitary infrastructures and the decline of mortality in Germany, 1877–1913 The Economic History Review 2020 73 3 730 757 10.1111/ehr.12942
Gutberlet, T. (2013), Railroads and the regional concentration of industry in Germany 1861 to 1882, mimeo, Rensselaer Polytechnic Institute.
Hornung E Railroads and growth in Prussia Journal of the European Economic Association 2015 13 4 699 736 10.1111/jeea.12123
Keller, W. und C. H. Shiue (2014), The link between fundamentals and proximate factors in development, NBER Working Paper, 18808, National Bureau of Economic Research.
Nelson R R Phelps E S Investment in Humans, Technological Diffusion, and Economic Growth American Economic Review 1966 56 1/2 69 75
Schueler, R. (2016), Centralized Monitoring, Resistance, and Reform Outcomes. Evidence from School Inspections in Prussia, ifo Working Paper, 223.
Ziegler, D. (1996), Eisenbahnen und Staat im Zeitalter der Industrialisierung: die Eisenbahnpolitik der deutschen Staaten im Vergleich, Franz Steiner Verlag.
